# A peroxidase-like magneto-gold nanozyme AuNC@Fe_3_O_4_ with photothermal effect for induced cell apoptosis of hepatocellular carcinoma cells *in vitro*

**DOI:** 10.3389/fbioe.2023.1168750

**Published:** 2023-03-23

**Authors:** Xinglong Shi, Jifa Liu, Guannan Wang

**Affiliations:** ^1^ College of Medical Engineering & the Key Laboratory for Medical Functional Nanomaterials, Jining Medical University, Jining, China; ^2^ Cheeloo College of Medicine, Shandong University, Jinan, China

**Keywords:** POD-like, magneto-gold, nanozyme, photothermal effect, cell apoptosis, hepatocellular carcinoma

## Abstract

Hepatocellular carcinoma (HCC) is one of the most commonly diagnosed and malignant cancers worldwide. Conventional therapy strategies may not completely eradicate the tumor and may cause side effects during treatment. Nano-catalytic therapy, as a novel strategy, has attracted a great deal of attention. This study aimed to synthesize a multifunctional magneto-gold nanozyme AuNC@Fe_3_O_4_ and evaluate its anti-cancer potential in HepG2 cells *in vitro*. The characteristics of AuNC@Fe_3_O_4_ were assessed using a transmission electron microscope, dynamic light scattering, and energy-dispersive X-ray. The photothermal performance and peroxidase (POD)-like activity of AuNC@Fe_3_O_4_ were detected, using thermal camera and ultraviolet-visible spectrophotometer, respectively. The anti-cancer potential of AuNC@Fe_3_O_4_ was examined using cell counting kit-8, live/dead cell staining, and apoptosis analysis. Further research on HepG2 cells included the detection of intracellular reactive oxygen species (ROS) and lysosomal impairment. We observed that the AuNC@Fe_3_O_4_ had a small size, good photothermal conversion efficiency and high POD-like activity, and also inhibited cell proliferation and enhanced cell apoptotic ability in HepG2 cells. Furthermore, the AuNC@Fe_3_O_4_ enhanced ROS production and lysosomal impairment *via* the synergistic effect of photothermal and nano-catalytic therapies, which induced cell death or apoptosis. Thus, the magneto-gold nanozyme AuNC@Fe_3_O_4_ may offer a potential anti-cancer strategy for HCC.

## 1 Introduction

Data from Global Cancer Incidence, Mortality and Prevalence 2020 revealed that liver cancer was the sixth commonly diagnosed and the third lethal cancer worldwide (Sung et al., 2021). China accounted for 23.7% and 30% of the global morbidity and mortality from liver cancer, respectively (Ferlay et al., 2021). It was predicted that between 2020 and 2040, there would be a 55% increase in the number of new cases of liver cancer per year, and the percentage of people who would die from the disease in 2040 would be more than 56.4% of those in 2020 (Rumgay et al., 2022). Primary liver cancer can be classified into three types: cholangiocarcinoma, hepatocellular carcinoma (HCC), and a combination of the two, with HCC accounting for approximately 90% of all cases (Llovet et al., 2016). HCC progression is influenced by several risk factors, such as alcohol abuse, smoking, toxic chemicals, and hepatitis virus (especially for HBV) infections (Yang et al., 2019). Owing to the absolute number of HBV-infected populations (Liu et al., 2016), the mortality rate of HBV-related liver cancer was consistently higher than the global level (Liu et al., 2019), which increased the burden of HCC in China.

In most cases, conventional treatments, such as surgery, radiation, and chemotherapy, do not completely eradicate the tumor and may cause side effects during treatment, such as cancer palindromia and drug resistance (Zhu et al., 2016; Xu et al., 2019; Raoul and Edeline, 2020; Liu and Song, 2021). For example, the surgery was initially considered to be used for patients with early-stage HCC; However, over 50% of patients experienced a recurrence within a year following surgery (Gil et al., 2015; Weber et al., 2015). Sorafenib was an option for patients with advanced-stage cancer, however, it was only effective in less than a third of them and caused drug tolerance or cytotoxicity (Llovet et al., 2008; Cheng et al., 2009; Bruix et al., 2012; Anwanwan et al., 2020). Besides surgery and chemotherapy, radiation therapy is a non-invasive and local ablative treatment approach to kill cancer cells. However, the efficiency of radiation therapy is easily limited by radioresistance, due to the DNA damage response and cell cycle checkpoints activation (Yoon and Seong, 2014; Wahl et al., 2016; Sun et al., 2020). Although the traditional strategies of HCC control the growth of HCC and prolong the survival time of patients, it still cannot satisfy their needs. Thus, it is necessary to discover a more efficient treatment approach to improve the quality of life for patients.

In recent years, nano-catalytic therapy, as a new tumor treatment strategy, has attracted the attention of an increasing number of researchers. Nanozymes are nanomaterials that catalyze chemical reactions of substrates under physiological states, obeying the patterns of enzyme kinetics (Wei et al., 2021). In 2007, Yan’s team was the first to report that magnetic nanoparticles Fe_3_O_4_ possessed peroxidase (POD)-like activity, and proposed the concept of nano-catalysis (Gao et al., 2007). Furthermore, Shi *et al.* innovatively paved the way for further applications of nanoparticles in tumor nano-catalytic therapy, by disrupting the Fenton reaction that induced H_2_O_2_ disproportionation for •OH generation (Zhang et al., 2016). Currently, nano-catalytic therapy and photothermal therapy (PTT) are frequently employed in the treatment of tumors. The integration of PTT and nano-catalytic therapy has contributed to improving their cancer therapy efficiency. For instance, hyperthermia promoted the enzymatic activity of Fe_3_O_4_ nanozyme to generate more •OH, and simultaneously, •OH heightened the therapeutic impact of PTT (Wu et al., 2019; Zuo et al., 2022). It has also been reported that the Fe_3_O_4_@ZIF-8/GOx@MnO_2_ hybrid nanozyme can enhance the efficiency of nanoparticles in anti-tumor therapy by combining multiple therapeutics (Zhang et al., 2021b).

Fe_3_O_4_ and Au nanoparticles, as is well known, demonstrated the unique characteristics of a high photothermal effect and POD-like activity (Zeng et al., 2013; Vallabani et al., 2017; Ghosh et al., 2022; Huang et al., 2022). Encouraged by the aforementioned description, we wonder if AuNC@Fe_3_O_4_ which has been employed as magnetic resonance imaging/com-puterized tomography multimodal imaging contrast agents of cancer owing to their high relaxivity value and excellent contrast enhancement (Wang et al., 2016b), also retains the photothermal and catalytic ability, or is beneficial to cancer therapy.

In this study, we synthesized multifunctional magneto-gold nanozyme AuNC@Fe_3_O_4_ and evaluated their anti-cancer ability in HCC cells *in vitro*. The AuNC@Fe_3_O_4_ exhibited high photothermal effect and POD-like activity. The results also reflected the influence of AuNC@Fe_3_O_4_ on engendering cell death and apoptosis. Furthermore, the synergistic effect of PTT and nano-catalytic therapy on reactive oxygen species (ROS) and lysosomal impairment in HepG2 cells were also studied.

## 2 Materials and methods

### 2.1 Materials and reagents

Ferric slat, gold (III) chloride (HAuCl4), other reagents related to AuNC@Fe_3_O_4_ synthesis and 3,3′,5,5′-Tetramethylbenzidine (TMB) were purchased from Sigma, Inc. (St. Louis, United States). H_2_O_2_ solution and different pH buffer solutions (pH = 2, 3, 4, 5, 6, 7, 8, and 9) were bought from Aladdin (Shanghai, China). Human umbilical vein endothelial cells (HUVEC), human HCC cell lines (HepG2 cells) and the specific culture mediums for the two cell lines were purchased from Procell (Wuhan, China). Cell Counting Kit-8 (CCK-8) was obtained from Sangon Biotech (Shanghai, China). 2′,7′-Dichlorodihydrofluorescein diacetate (DCFH-DA) was obtained from MedChemExpress (New Jersey, United States). Calcein-AM/propidium iodide (PI) kit, Annexin V-FITC apoptosis detection kit, Lyso-Tracker Red kit and Hoechst 33342 staining solution were purchased from Beyotime. Inc. (Shanghai, China).

### 2.2 AuNC@Fe_3_O_4_ synthesis

The AuNC@Fe_3_O_4_ was synthesized according to previous methods (Wang et al., 2016b). AuNC was initially synthesized and coated with poly (vinyl pyrrolidone) (PVP). Subsequently, PVP was replaced with 2-aminoethanethiol, and AuNC was transformed into AuNC-NH2 for interacting with carboxyl group functionalized Fe_3_O_4_ nanoparticles. The ultra-small Fe_3_O_4_ particles were prepared. To produce Fe_3_O_4_-COOH, ferric slats were vigorously stirred in pre-prepared polymer poly (acrylic acid) (PAA) solution. N-(3-Dimethylaminopropyl)-N-ethylcarbodiimide and N-hydroxysuccinimide activated the Fe_3_O_4_-COOH, which then reacted with AuNC-NH_2_ to generate AuNC@Fe_3_O_4_. The AuNC@Fe_3_O_4_ was centrifugated, washed with ethanol and water, and then dispersed in ddH_2_O with different concentrations for further experiments.

### 2.3 AuNC@Fe_3_O_4_ characterization

The size of AuNC or AuNC@Fe_3_O_4_ nanoparticles was analyzed using a transmission electron microscope (TEM). Dynamic light scattering (DLS) was applied to detect hydrodynamic particle diameter and intensity of AuNC@Fe_3_O_4_ nanoparticles on a Malvern Zetasizer NANO ZS. Energy-dispersive X-ray (EDX) was utilized to analysis the element of AuNC@Fe_3_O_4_ nanoparticles on a FEI TECNAI G20 high-resolution TEM.

### 2.4 AuNC@Fe_3_O_4_ photothermal performance *in vitro*

To investigate the photothermal effect of the magneto-gold nanoparticles, First, 200 μl of AuNC@Fe_3_O_4_ solution with distinct concentrations (0, 50, 100, 200, 300, 400, and 500 μg/ml) was exposed to 808 nm laser at 1.0 W for 720 s; Second, 200 μl of AuNC@Fe_3_O_4_ solution with concentration of 50 μg/ml was exposed to 808 nm laser at different powers (1.0, 1.2, and 1.4 W) for 720 s. The thermal image and temperature change were recorded at different times by an infrared (IR) thermal camera (Fotric 220). As a control, ddH_2_O was irradiated under the same conditions.

To investigate the photothermal stability of the magneto-gold nanoparticles, AuNC@Fe_3_O_4_ aqueous solution (500 μg/ml) was irradiated under 808 nm laser at 1.0 W for 420 s, then the irradiation was turned off. After that, the temperature was further measured for another 360 s. The experiment was then repeated four more times. The thermal image and temperature change were recorded at different times by the IR thermal camera (Fotric 220). As a control, ddH_2_O was irradiated under the same operation.

To evaluate the photothermal conversion efficiency of AuNC@Fe_3_O_4_, the data from the cooling periods were calculated, according to previous report (Ren et al., 2015). Briefly, when the system reached energy balance, the equation was:

$$\underset{i}{\sum }m_{i}C_{p,i}\frac{dT}{dt}=Q_{{AuNC@Fe}_{3}O_{4}}+Q_{s}-Q_{loss}$$ (1)

where C*p* and m were the heat capacity and mass of AuNC@Fe_3_O_4_ solution, respectively. *T* was the temperature of AuNC@Fe_3_O_4_ solution. $Q_{{AuNC@Fe}_{3}O_{4}}$ represented energy absorbed by AuNC@Fe_3_O_4_ nanoparticles. $Q_{s}$ represented the energy absorbed by ddH_2_O. $Q_{loss}$ was the heat lost to the surroundings.

The equation for $Q_{{AuNC@Fe}_{3}O_{4}}$ was:

$$Q_{{AuNC@Fe}_{3}O_{4}}=I\left(1-10^{{-A}_{\lambda }}\right)\eta$$ (2)

where *I* represented the laser power density, $A_{\lambda }$ denoted the absorbance of AuNC@Fe_3_O_4_ solution under 808 nm in a 96-well plate, and η represented its photothermal conversion efficiency.

The equation for $Q_{loss}$ was

$$Q_{loss}=hA\Delta T$$ (3)

where *A* was the surface area of the container, *h* denoted the heat transfer coefficient; *ΔT* represented the temperature changes, expressed as T-T_*surr*_ (where T and T_*surr*_ represent the solution and surrounding air temperature, respectively).

When heating ddH_2_O, the heat input and output reached energy balance at the maximum steady-state temperature, therefore the equation for $Q_{s}$ was:

$$Q_{s}=Q_{loss}=hA{\Delta T}_{\max,H_{2}O}$$ (4)

where ${\Delta T}_{\max,H_{2}O}$ was the temperature changes of ddH_2_O.

When the system reached its maximum balanced temperature, the energy input (the heat absorbed by AuNC@Fe_3_O_4_ and ddH_2_O) was equal to the heat lost into the surrounding, and the equation could be:

$$Q_{{AuNC@Fe}_{3}O_{4}}+Q_{s}=Q_{loss}=hA\Delta T_{\max,mix}$$ (5)

where $\Delta T_{\max,mix}$ was the changed temperature of the AuNC@Fe_3_O_4_ solution.

According to Eqs 2, 4, 5, η was:

$$\eta =\frac{hA\Delta T_{\max,mix}-hA{\Delta T}_{\max,H_{2}O}}{I\left(1-10^{{-A}_{\lambda }}\right)}=\frac{hA\left(\Delta T_{\max,mix}-{\Delta T}_{\max,H_{2}O}\right)}{I\left(1-10^{{-A}_{\lambda }}\right)}$$ (6)

To calculate the unknown hA , θ was introduced, and could be expressed as following:

$$\theta =\frac{\Delta T}{{\Delta T}_{\max}}$$ (7)

Adding Eq. 7 into Eq. 1, the new equation could be:

$$\frac{d\theta }{dt}=\frac{hA}{\underset{i}{\sum }m_{i}C_{p,i}}\left(\frac{Q_{{AuNC@Fe}_{3}O_{4}}+Q_{s}}{hA\Delta T_{\max}}-\theta \right)$$ (8)

During the cooling period, the $Q_{{AuNC@Fe}_{3}O_{4}}+Q_{s}$ = 0 in Eq. 8 was:

$$dt=-\frac{\underset{i}{\sum }m_{i}C_{p,i}}{hA}\frac{d\theta }{\theta }$$ (9)

which could be changed as following:

$$t=-\frac{\underset{i}{\sum }m_{i}C_{p,i}}{hA}\ln\theta$$ (10)

where $\frac{\underset{i}{\sum }m_{i}C_{p,i}}{hA}$ was calculated by time versus -ln(θ) plot. Since the mass of AuNC@Fe_3_O_4_ (1 × 10^−7^ kg) was relatively small when compared to that of ddH_2_O (m = 2 × 10^−4^ kg), its m and Cp were neglected. The value of *hA* was then calculated using $m_{H_{2}O}$ of 2 × 10^−3^ kg; $C_{p,H_{2}O}$ of 4.2 × 10^3^ J/(Kg·°C). Furthermore, the η of AuNC@Fe_3_O_4_ was determined by substituting the value of *hA* and other parameters into Eq. 6. The values of other parameters were as follows: *I* = 2.3 W/cm^2^, $A_{\lambda }$ = 0.105, $\Delta T_{\max,mix}$ = 25.3, and ${\Delta T}_{\max,H_{2}O}$ = 0.1.

### 2.5 POD-like activity assay

To evaluate the catalytic properties of AuNC@Fe_3_O_4_, AuNC@Fe_3_O_4_ (final concentration: 0, 5, 10, 20, 50, and 100 μg/ml), TMB (final concentration: 0.4 mM), and H_2_O_2_ (final concentration: 50 μM) was added into a final volume of 500 μl of phosphate-buffered saline (PBS) solution. The absorbance of the buffer was measured using an ultraviolet-visible (UV-vis) spectrophotometer at 500–800 nm. The POD-like activity assay of AuNC@Fe_3_O_4_ at varying pH levels (pH = 2, 3, 4, 5, 6, 7, 8, and 9) was performed in the presence of H_2_O_2_ and TMB in PBS solution, and the absorbance at 652 nm was detected by a microplate reader.

### 2.6 POD-like catalytic kinetic determination

When TMB was used as a substrate, the AuNC@Fe_3_O_4_ (final concentration: 50 μg/mL), TMB (final concentration: 0.0, 0.2, 0.4, 0.6, and 0.8 mM), and H_2_O_2_ (final concentration: 50 μM) was added into a final volume of 100 μl of PBS solution. The absorbance at 652 nm was detected by a microplate reader.

When H_2_O_2_ was used as a substrate, the AuNC@Fe_3_O_4_ (final concentration: 50 μg/ml), H_2_O_2_ (final concentration: 0, 10, 20, 30, 40, 50, 60, 70, and 80 μM) and TMB (final concentration: 0.4 mM) was added into a final volume of 100 μl of PBS solution. The absorbance at 652 nm was detected by a microplate reader.

Based on Michaelis-Menten Eq. 11 and saturation curve, the V_max_ and Michaelis-Menten constant could be calculated,

$$\frac{1}{V}=\frac{K_{m}}{V_{\max}}\frac{1}{\left[s\right]}+\frac{1}{V_{\max}}$$ (11)

and the *V* was calculated using Eq. 12:

$$V=\frac{A}{\left(b\times {Ɛ}_{652\;nm}\times t\right)}$$ (12)

where *A* was the absorbance of the reaction system at 652 nm. t = 600 s, which was the reaction time. b = 0.3125 cm, which was the light path in the reaction solution, and ${Ɛ}_{652\;nm}$ = 39,000 M^−1^ cm^−1^ (Dashtestani et al., 2019).

### 2.7 Cell viability assay

The HepG2 and the HUVEC cells were cultured to assess the cytotoxicity of AuNC@Fe_3_O_4_ through CCK-8 assay. 4000 of cells were cultured at 96-well plate well overnight at 37°C in a humidified incubator with 5% CO_2_. Subsequently, 100 μl of fresh medium with distinct concentrations of AuNC@Fe_3_O_4_ (0, 10, 20, 30, 40, and 50 μg/ml) was changed and cultured for 24 h. The CCK-8 solution (final volume: 10 μl) was added into 100 μl of medium, and incubated for 2 h. Then, the absorbance of medium was detected at 450 nm.

### 2.8 Live/dead cell staining assay

HepG2 cells were cultured overnight in a 12-well plate with 500 μl of culture medium. The cells were then treated with PBS or AuNC@Fe_3_O_4_ (50 μg/ml) for 12 h. Then, the cells were cultured for an additional 12 h after either being irradiated by an 808 nm laser for 5 min at 1.4 W or not. The culture medium was then removed, and cells were washed once with PBS and incubated with 500 μl stain solution for 15 min. Finally, the cells were washed thrice with PBS and photographed by an inverted fluorescence microscope.

### 2.9 Apoptosis analysis

To investigate the ability of AuNC@Fe_3_O_4_ for inducing cell apoptosis, HepG2 cells were quantitatively detected by a flow cytometer. The cells were initially seeded into a 6-well plate and treated under different conditions for 24 h. They were collected with 0.25% trypsin and washed thrice with ice-cold PBS. Subsequently, these cells were resuspended in 195 μl of binding buffer. Ten microliters of PI and 5 μl of Annexin V-FITC were added, and the mixture was incubated for 20 min at room temperature, and cells were detected by flow cytometer.

### 2.10 Intracellular ROS detection

The intracellular POD-like catalytic ability of AuNC@Fe_3_O_4_ was detected using DCFH-DA. Except for an additional 4 h of culture, the method used for the laser-irradiated groups was similar to the treatment described above. Furthermore, 1 ml of PBS with DCFH-DA (5 μM) was added, and the mixture was incubated for another 30 min at 37°C in a humidified incubator with 5% CO_2_. The wells were then washed thrice with PBS to remove the excess dye and photographed by an inverted fluorescence microscope.

### 2.11 Lysosomal impairment assay

After treatment, lysosomes and cell nuclei were stained with Lyso-Tracker Red and Hoechst 33342, respectively, according to the manufacturer’s instructions. Subsequently, an inverted fluorescence microscope was used to capture images of cells.

### 2.12 Statistical analysis

Statistical analysis was achieved by GraphPad Prism version 8 (GraphPad Software, United States). Results were represented as mean ± standard deviation. The student t-test was used to compare the means of multiple groups. The statistical significances were as follows: * 0.01 < *p* < 0.05, ** 0.001 < *p* < 0.01, and ***p < 0.001.

## 3 Results and discussion

### 3.1 Synthesis and characterization of AuNC@Fe_3_O_4_

**FIGURE 1:**
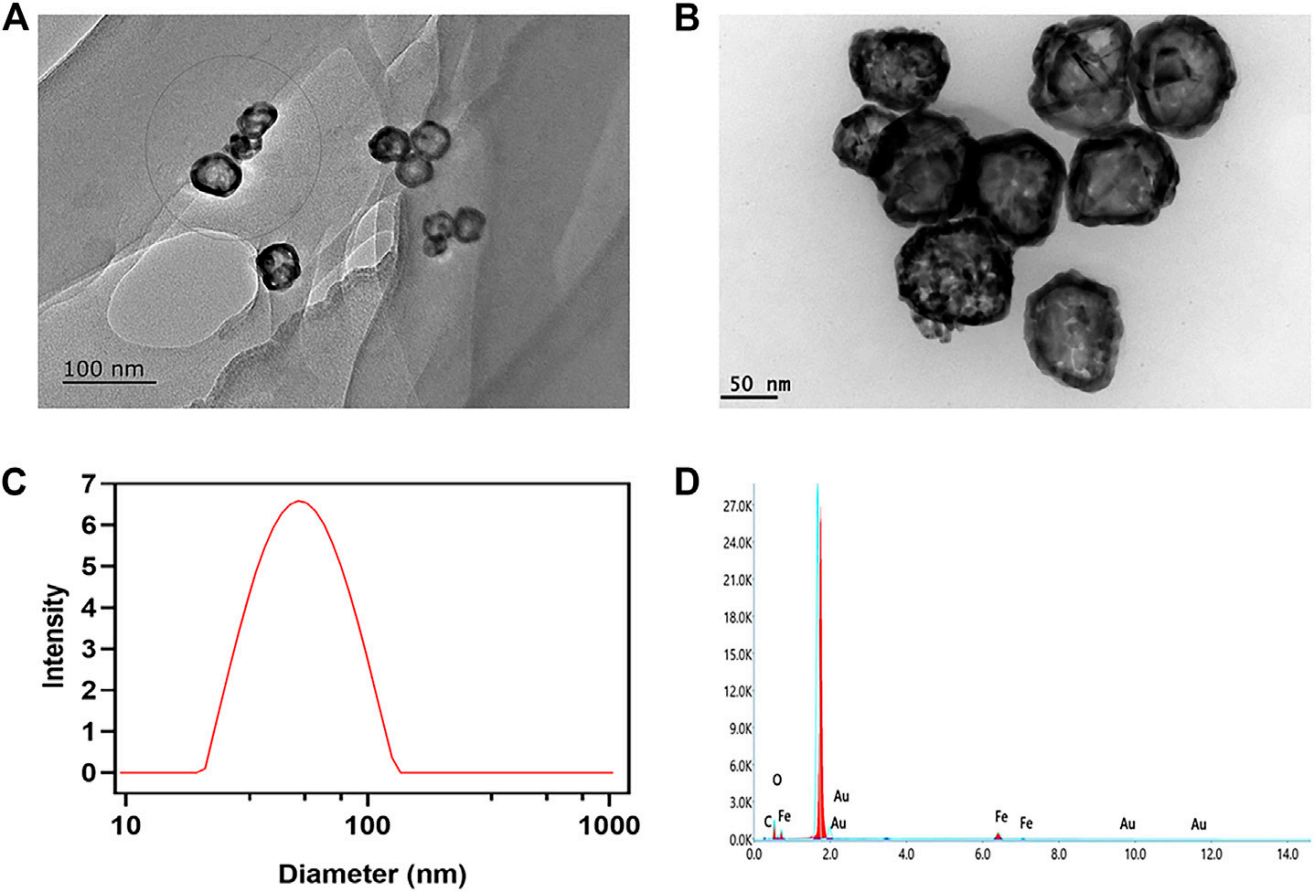
Characterization of AuNC@Fe_3_O_4._ **(A)** Transmission electron microscope (TEM) images of AuNC. Scale bar: 100 nm. **(B)** TEM images of AuNC@Fe_3_O_4_. Scale bar: 50 nm. **(C)** Dynamic light scattering (DLS) result of AuNC@Fe_3_O_4_. **(D)** Energy-dispersive X-ray (EDX) result of AuNC@Fe_3_O_4_.

The structure and characteristics of AuNC and AuNC@Fe_3_O_4_ were determined by TEM. The results demonstrated that the diameter of AuNC and AuNC@Fe_3_O_4_ were 25–40 and 50–100 nm, respectively, with high uniformity and no agglomeration (Figures 1A, B). DLS was used to confirm the size of AuNC@Fe_3_O_4_, and the average hydrodynamic size distribution of these nanoparticles was approximately 55 nm (Figure 1C). The increase in the hydrodynamic size might be owing to the attachment of Fe_3_O_4_ to the surface of the AuNC. Elemental mapping analysis revealed the presence of the atoms Au, Fe and O, proving that AuNC@Fe_3_O_4_ was successfully formed (Figure 1D; Table 1). The “-CO-NH-”, that came from the reaction of Fe_3_O_4_-COOH and AuNC-NH_2_ and the carbon-coated brace used during sample preparation or analysis might have contributed to the existence of C element that was also present (Phongtongpasuk et al., 2016).

**TABLE 1:** The statistics of elements analysis for AuNC@Fe_3_O_4_ by energy-dispersive X-ray (EDX).

Compound	Element	Weight (%)
AuNC@Fe_3_O_4_	Au	23.1
	Fe	29.4
	O	42.4
	C	5.1

### 3.2 Photothermal performance of AuNC@Fe_3_O_4_

**FIGURE 2:**
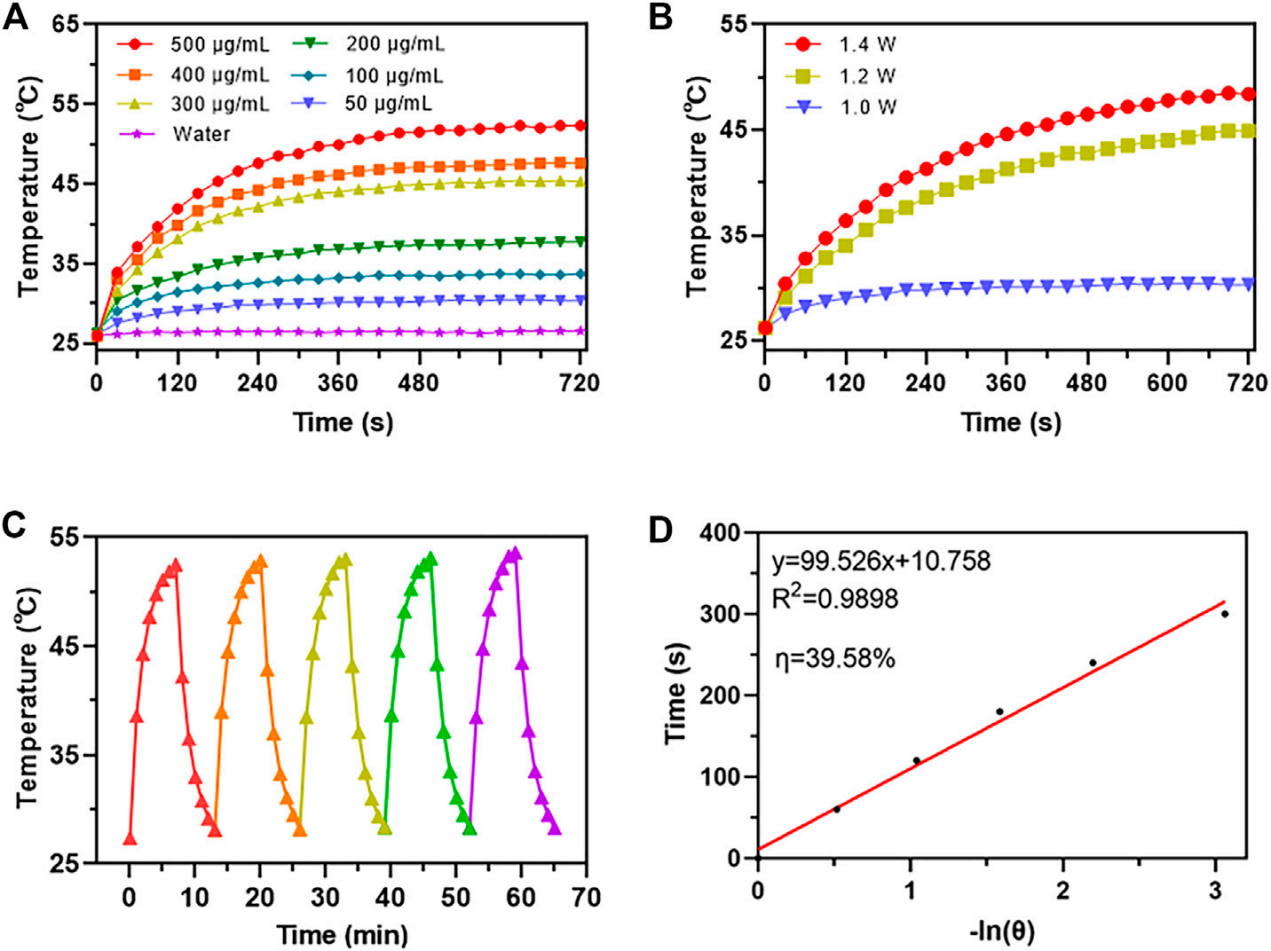
Photothermal performance analysis of AuNC@Fe_3_O_4_. **(A)** Temperature change curves of water and AuNC@Fe_3_O_4_ aqueous solution after different treatments **(B)** Temperature change curves of AuNC@Fe_3_O_4_ after different treatments **(C)** Photostability of AuNC@Fe_3_O_4_ solution under irradiation for five cycles. **(D)** Time versus -ln(θ) plot of the AuNC@Fe_3_O_4_ solution.

The thermal camera was used to investigate the photothermal conversion capabilities of AuNC@Fe_3_O_4_. The temperature changes of AuNC@Fe_3_O_4_ solution with different concentrations under 808 nm laser irradiation at 1.0 W for 360 s were recorded. As depicted in Figure 2A, the temperature of the solution increased in a concentration- and time-dependent pattern. For example, the temperature of different concentrations of AuNC@Fe_3_O_4_ solution reached steady statue at 8 min. The temperature of AuNC@Fe_3_O_4_ solution (500 μg/ml) was changed significantly from 25.9°C to 52.3°C compared with the neglected increase in that of ddH_2_O (from 26.0°C to 26.5°C), indicating the good photothermal response of AuNC@Fe_3_O_4_. For further investigation, the AuNC@Fe_3_O_4_ solution (50 μg/ml) was irradiation at different powers (1.0, 1.2, and 1.4 W). The laser power was increased from 1.0 to 1.4 W, which resulted in a significant increase in the temperature of the AuNC@Fe_3_O_4_ solution. A temperature of 45.3°C was achieved after 10 min of 808 nm laser irradiation at 1.4 W (Figure 2B). PTT, a promising cancer treatment strategy, converts light energy into heat to generate an area of hyperthermia, where tissues can be exposed to high temperatures (from 42°C to 45°C), which can damage or kill tumor cells (Tchouagué et al., 2019; Qu et al., 2022). The results of Figures 2A, B suggested a potential application of AuNC@Fe_3_O_4_ in anti-tumor.

Additionally, five cycles of the “On and Off” model were used to measure the temperature curve of the AuNC@Fe_3_O_4_ solution to assess its photothermal stability. The AuNC@Fe_3_O_4_ showed excellent photothermal stability since the temperature was raised to 52.9 °C and there was no reduction in the temperature rise following laser irradiation during the five cycles (Figure 2D).

Moreover, the average of the data from the five cooling periods was used to get the photothermal conversion efficiency (η) of AuNC@Fe_3_O_4_. The plot of the time value and −ln (θ) was displayed in Figure 2E, and its slope was 99.526. Using Eqs 6, 10, the η of AuNC@Fe_3_O_4_ was calculated to be 39.58%, which was similar with or higher than the PPT reagents previously reported, such as, EA-Fe@BSA NPs (31.2%) (Tian et al., 2020), Fe_3_O_4_@Carbon@Platinum-Chlorin e6 (28.28%) (Xu et al., 2022b), Au nanorods (22%) (Zeng et al., 2013), Au nanoshells (13%) (Hessel et al., 2011), PANi@Au (40.4%) and Au nanoparticles (21.7%) (Zhang et al., 2021a).

Collectively, these findings suggested that AuNC@Fe_3_O_4_ exhibited good photothermal conversion and photothermal stability, which implied a promising application in PTT for tumors.

### 3.3 POD-like activity of AuNC@Fe_3_O_4_

**FIGURE 3:**
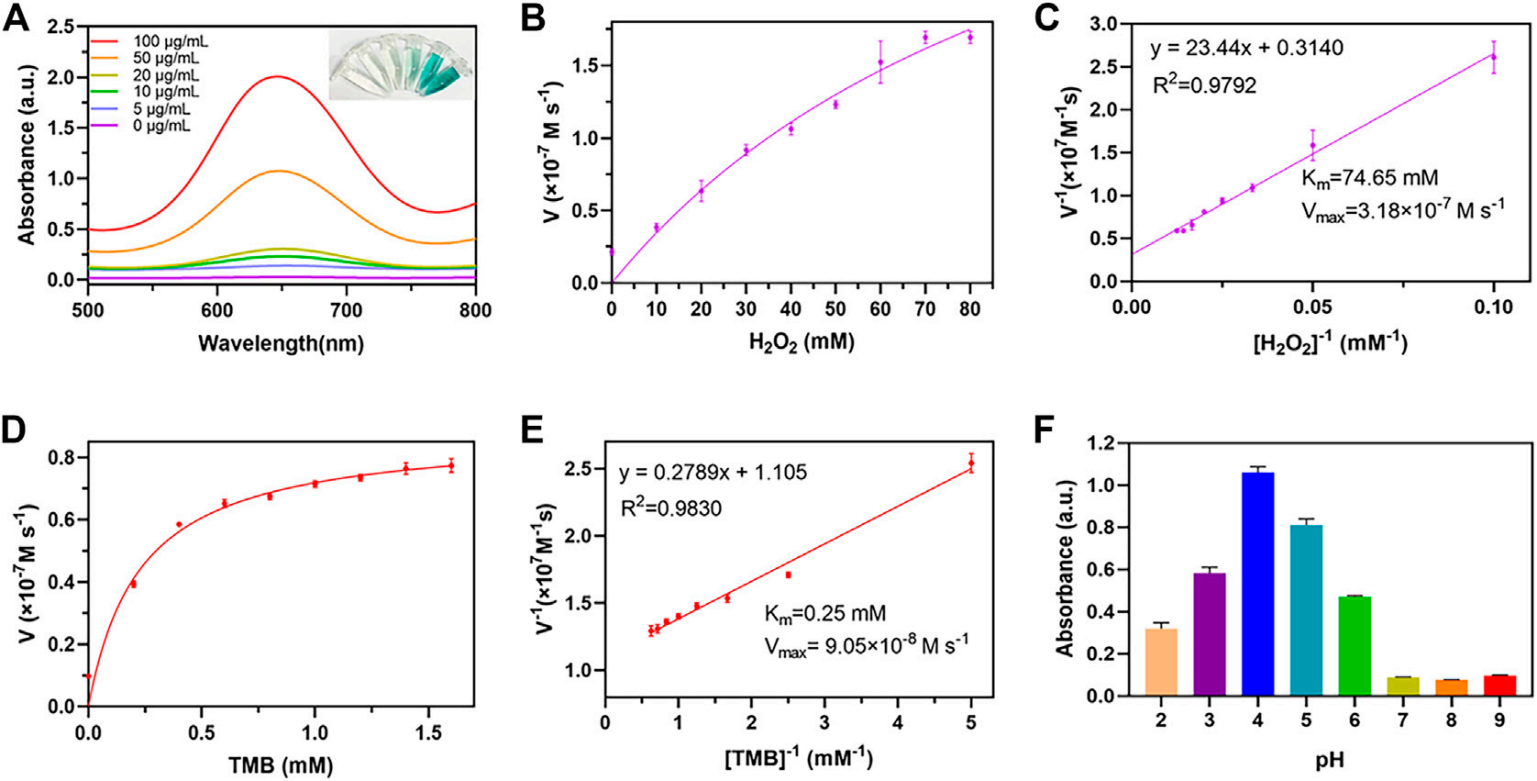
POD-like activity assay of AuNC@Fe_3_O_4_. **(A)** Ultraviolet-visible (UV-vis) absorption spectra of the reaction system with different concentrations. **(B)** Michaelis-Menten curve of AuNC@Fe_3_O_4_ for H_2_O_2_. **(C)** Lineweaver-Burk plotting of AuNC@Fe_3_O_4_ for H_2_O_2_. **(D)** Michaelis-Menten curve of AuNC@Fe_3_O_4_ for TMB. **(E)** Lineweaver-Burk plotting of AuNC@Fe_3_O_4_ for TMB. **(F)** The absorbance of the reaction system at 652 nm under different pH values.

It was reported that Au and Fe_3_O_4_ nanoparticles demonstrated POD-like enzyme activity (Zandieh and Liu, 2021), therefore it was necessary to investigate whether the AuNC@Fe_3_O_4_ possessed similar characteristics. The peroxidase mimicking activity of AuNC@Fe_3_O_4_ was validated by TMB. TMB could be oxidized to blue oxTMB by •OH and detected at 652 nm, using UV-vis spectrophotometer (Zhu et al., 2022). As presented in Figure 3A, the groups with different concentrations of AuNC@Fe_3_O_4_ had varying absorbance intensities at 652 nm. The group of AuNC@Fe_3_O_4_ with 100 μg/ml showed the strongest signal at 652 nm, followed by the group with 50 μg/ml. The intensity of absorbance tested with H_2_O displayed no peak at 652 nm. These findings, which indicated that the AuNC@Fe_3_O_4_ possessed POD-like enzyme activity, were further verified by the inset digital photos (Figure 3A). To further confirm the POD-like enzyme specificity of AuNC@Fe_3_O_4_, the UV-vis absorption spectra of the reaction system with varying conditions was collected. It was observed from Supplementary Figure S1 that absorbance peak of the AuNC@Fe_3_O_4_+TMB or AuNC@Fe_3_O_4_+H_2_O_2_ group was negligible. In the absence of AuNC@Fe_3_O_4_, the TMB + H_2_O_2_, TMB or H_2_O_2_ group showed no significant absorbance peak at 652 nm, which was consistent with the AuNC@Fe_3_O_4_ only group. The results suggested that, except AuNC@Fe_3_O_4_, other components in the reaction system could hardly catalyzed the conversion of H_2_O_2_ to •OH and oxidized TMB, which indicated AuNC@Fe_3_O_4_ exhibited a specific activity of POD-like enzyme.

Kinetic parameters were analyzed to quantitate the POD-like activity of AuNC@Fe_3_O_4_ using the initial rate method (Gao et al., 2017). First, the absorbance of the system was measured, while varying the concentrations of H_2_O_2_ concentrations from 0 to 80 mM and maintaining a TMB concentration of 0.4 mM (Supplementary Figure S2). Second, the velocity of reaction was calculated according Eq. 12 and the plot was consistent with traditional Michaelis-Menten curve (Figure 3B), which demonstrates that the catalytic reaction rate increased with the growth of substrate concentration and achieved steady state at high concentrations (Huang et al., 2022). Third, after Lineweaver-Burk fitting, the enzyme kinetic parameters, such as Michaelis-Menten constants (K_m_) was calculated to be 47.65 mM and the maximum reaction velocity (V_max_) was 3.18 × 10^−7^ M s^−1^ (Figure 3C). Forth, the absorbance of the solution was measured at 652 nm while varying TMB concentrations and maintaining H_2_O_2_ concentration as a constant (Supplementary Figure S3). Last, the K_m_ and V_max_ were 0.25 mM and 9.03 × 10^−8^ M s^−1^ respectively, and the results were presented in Figures 3D, E.

When the H_2_O_2_ was used as substrate, the velocity of AuNC@Fe_3_O_4_ was faster than that of Fe_3_O_4_ (Vallabani et al., 2017), and K_m_ value that was lower than that of Fe_3_O_4_ (Vallabani et al., 2017). Similarly, when the TMB was used as substrate, AuNC@Fe_3_O_4_ had a velocity that was faster than that of Au NRT, Au NC, Au NS, and horseradish peroxidase (Ghosh et al., 2022), and its value of K_m_ was also lower than those of them. In the catalytic reaction system, the K_m_ represents the affinity between the enzyme and substrates, and the lower the K_m_, the higher enzyme affinity (Jiang et al., 2018). Therefore, the results suggested that the catalytic ability and the affinity between AuNC@Fe_3_O_4_ nanozyme and substrates (such as TMB and H_2_O_2_) was stronger than that of Fe_3_O_4_ and Au nanoparticles. The following factors may contribute to the significant increase in POD-like activity of AuNC@Fe_3_O_4_ nanoparticles: the electronic structure of the interfaces between the Fe_3_O_4_ and Au, the synergistic effect, and polarization effects from Au to Fe_3_O_4_ (Lee et al., 2010; Sun et al., 2013; Wang et al., 2016a).

Considering the complex tumor microenvironment, such as hypoxia and weak acidity (Li et al., 2020; Zhao et al., 2021), it was unclear whether AuNC@Fe_3_O_4_ exhibits POD-like enzyme activity even at low pH. At low pH values ranging from 2 to 6, the AuNC@Fe_3_O_4_ exhibited higher POD-like enzyme activity, and the optional pH was 4. When the pH was higher than 7, the POD-like enzyme activity was reduced dramatically (Figure 3F). The results hinted that AuNC@Fe_3_O_4_ might have significantly varied POD-like enzyme activity between distinct parts of normal (pH = 7.4) and cancer tissues (pH = 6.5), especially for lysosomes (pH = 4.5–5.5) and endosomes (pH = 5.5–6.8) (Kuppusamy et al., 2002; Wojtkowiak et al., 2011).

Overall, these findings provided evidence for the high POD-like catalytic activity of AuNC@Fe_3_O_4_ nanozyme and implied potential catalytic ability in tumor.

### 3.4 *In vitro* anti-tumor effect of AuNC@Fe_3_O_4_

**FIGURE 4:**
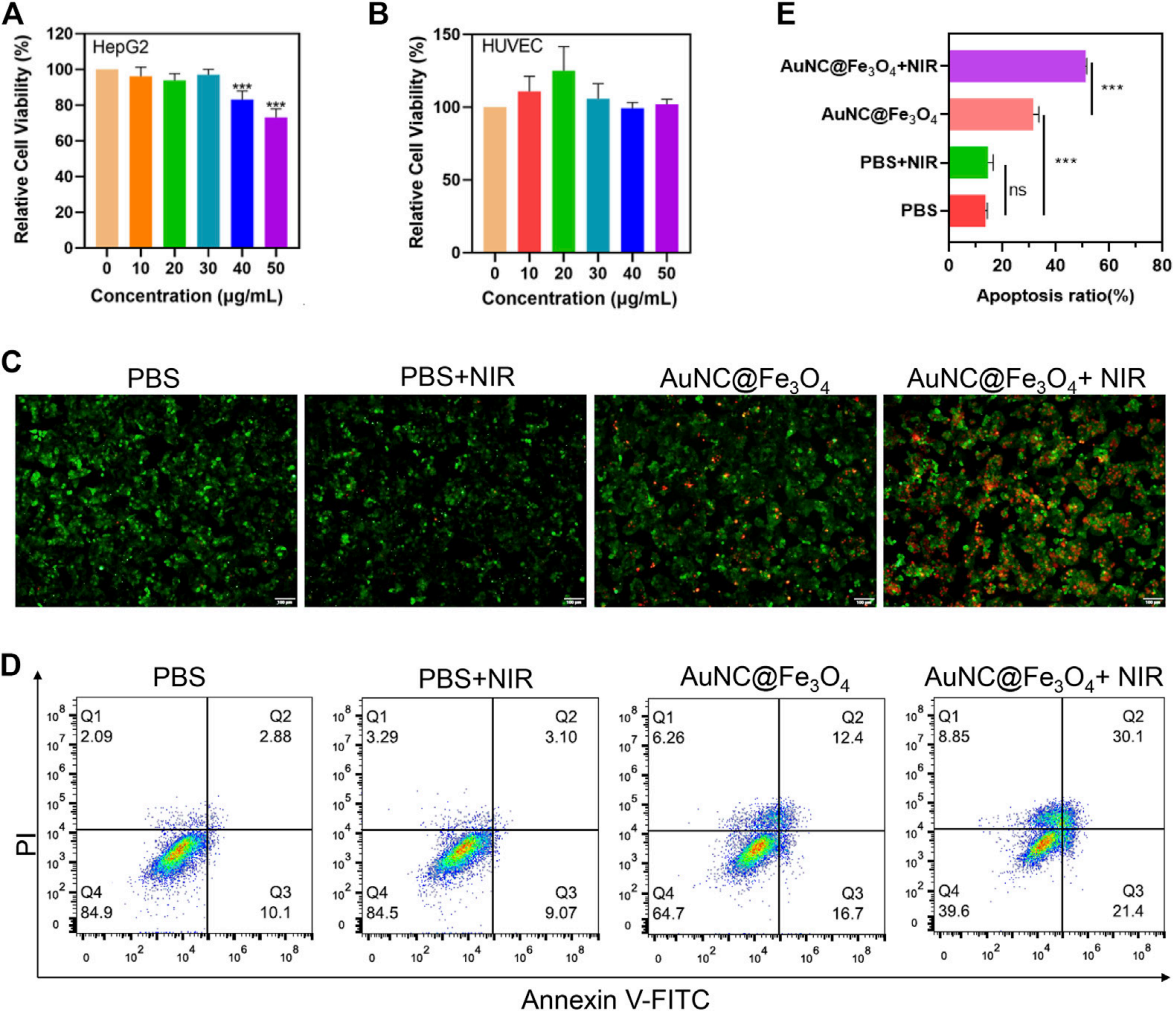
Evaluation for anti-tumor effect of AuNC@Fe_3_O_4_ *in vitro*. **(A)** Cell viability of HepG2 cells treated with AuNC@Fe_3_O_4_ for 24 h. **(B)** Cell viability of human umbilical vein endothelial cells (HUVEC) cells treated with AuNC@Fe_3_O_4_ for 24 h. **(C)** Calcein-Am/propidium iodide (PI) staining of HepG2 cells under different conditions. Scale bar: 100 μm. **(D)** Apoptosis analysis of HepG2 cells with different treatments. **(E)** The histogram results of apoptotic HepG2 cells derived from **(D)**. * 0.01 < *p* < 0.05, ** 0.001 < *p* < 0.01, and ***p < 0.001.

It is important to examine the biocompatibility of AuNC@Fe_3_O_4_ before performing further clinical applications. Therefore, HepG2 and HUVEC cells were incubated with AuNC@Fe_3_O_4_ at varying concentrations for 24 h to estimate the cytotoxicity using CCK-8 assay. Low concentrations of AuNC@Fe_3_O_4_ did not affect the survival rate of HepG2 cells; however, at 40 and 50 μg/ml, the viability of cells decreased to 77% and 60%, respectively (Figure 4A). In contrast, the viability of HUVEC cells was not drastically affected by AuNC@Fe_3_O_4_ after 24 h incubation at the varying treatments (Figure 4B). The findings indicated that AuNC@Fe_3_O_4_ was not toxic to normal cells at the concentration ranging from 0 μg/ml to 50 μg/ml and demonstrated good biocompatibility. The reason why AuNC@Fe_3_O_4_ showed more sensitive to HepG2 could be attributed to the fact that the pH of the tumor was lower than that of normal tissues (Kuppusamy et al., 2002; Wojtkowiak et al., 2011) and that the AuNC@Fe_3_O_4_ had higher POD-like enzyme activity in a lower pH reaction system, which meant it produced more •OH, which could be lethal to cells (Cui et al., 2018; Malfanti et al., 2022).

To explore the anti-tumor effect of AuNC@Fe_3_O_4_, the live/dead cell staining assay was utilized. There were nearly no dead cells in the PBS and PBS + NIR (near-infrared) groups; However, when the cells were treated with AuNC@Fe_3_O_4_ or AuNC@Fe_3_O_4_+NIR, the number of dead cells increased significantly, with the last group having the most cell death (Figure 4C). To further verify this result, the flow apoptosis assays of HepG2 cells with different conditions was conducted. As depicted in Figures 4D, E; Supplementary Table S1, the results indicated that approximately 51% apoptotic cells (Q2+Q3) were observed in the AuNC@Fe_3_O_4_+NIR group, which was greater than other groups.

The results revealed that AuNC@Fe_3_O_4_ displayed good biocompatibility, and the laser irradiation augmented the anti-tumor ability of AuNC@Fe_3_O_4_.

### 3.5 ROS and lysosomal impairment induced by AuNC@Fe_3_O_4_

**FIGURE 5:**
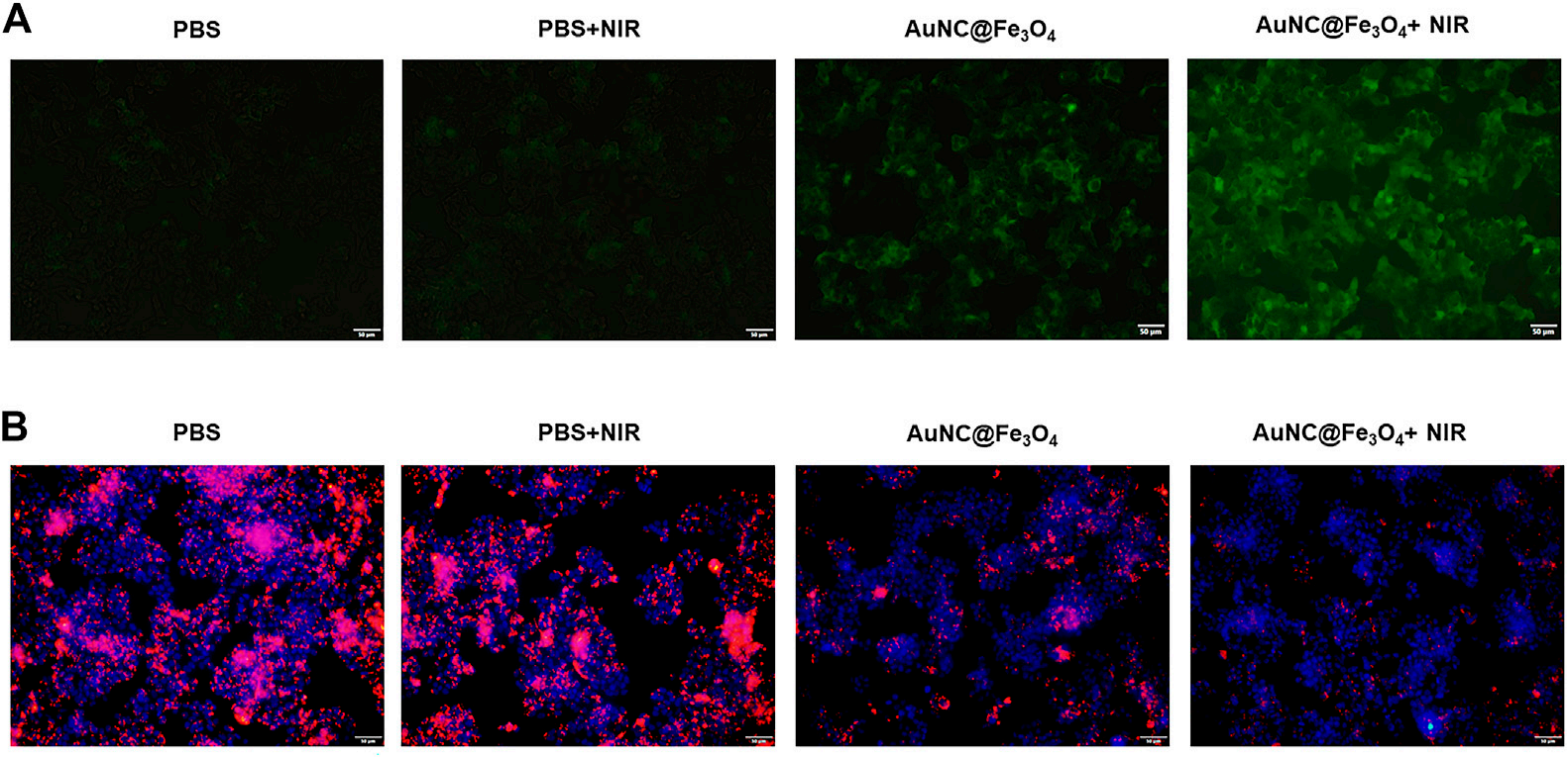
Analysis for synergistic effect of photothermal therapy (PTT) and catalytic activity of AuNC@Fe_3_O_4_. **(A)** Reactive oxygen species (ROS) detection of HepG2 cells with varying treatments. **(B)** Lysosomal impairment detection of HepG2 cells with varying treatments. Scale bar: 50 μm.

To confirm the synergistic effect of PTT and POD-like enzyme catalytic activity of AuNC@Fe_3_O_4_, the production of the ROS in HepG2 cells was validated using the DCFH-DA probe. As reported, DCFH-DA crossed the cell membrane and was subsequently oxidized to DCF with green fluorescence (Afri et al., 2004). It was evident from Figure 5A that the HepG2 cells incubated with AuNC@Fe_3_O_4_ exhibited a higher green fluorescence signal than PBS and PBS + NIR, indicating the ability of AuNC@Fe_3_O_4_ to effectively catalyze the conversion of intracellular H_2_O_2_ into •OH in cancer cells. Compared with the AuNC@Fe_3_O_4_ group, the signal of the AuNC@Fe_3_O_4_+NIR group was stronger. The similar result was collected by the detection of the absorbance intensities at 652 nm of the reaction system with or without NIR irradiation, using UV-vis spectrophotometer. We found the signal of reaction system with NIR irradiation was higher than that of group without irradiation (Supplementary Figure S4). The results confirmed that photothermal effect enhanced the POD-like enzyme catalytic activity of AuNC@Fe_3_O_4._

The phenomenon could be attributed to the localized surface plasmon resonance (LSPR), which was the collective oscillation of surface free electrons in metal nanoparticles under light irradiation, leading to local heating (also called photothermal effect) and hot carriers (such as hot electrons and hot holes). One hand, energy of hot electrons may transfer to local heating by electron-phonon interactions, causing a rise in temperature (Brongersma et al., 2015). Similar with natural enzymes, the catalytic ability of nanozymes could be enhanced by elevated temperature (Wang et al., 2021; Zhu et al., 2022). Another hand, hot electrons could be transferred from AuNPs to empty orbits of H_2_O_2_, and activated the H_2_O_2_ to generate •OH under NIR light irradiation (Wang et al., 2017; Xu et al., 2022a).

It was reported that increased ROS could disrupt normal structure of the lysosomes (Shyam et al., 2021); however, whether the AuNC@Fe_3_O_4_ could induce lysosomal impairment remained unknown. The fluorescence images (Figure 5B) demonstrate that the PBS alone and PBS + NIR groups had negligible effects on the lysosomal impairment and that there were more HepG2 cells with lysosomal impairment following incubation with AuNC@Fe_3_O_4_. As expected, the lysosomal signal was the weakest in the AuNC@Fe_3_O_4_ under laser irradiation group. The results confirmed the synergistic effect of PTT and POD-like enzyme catalytic activity of AuNC@Fe_3_O_4_ on lysosomal impairment. Additionally, lysosomal impairment may contribute to an increase in lysosomal membrane permeability, a decrease in lysosomal quantity, a disruption in lysosomal enzyme activities, an increase in ROS levels, and most importantly, the induction of cell apoptosis (Abulikemu et al., 2022).

This at least partly, explained why AuNC@Fe_3_O_4_ with or without laser irradiation could cause cell death or apoptosis.

## 4 Conclusion

In summary, this study aimed to synthesize magneto-gold nanozyme AuNC@Fe_3_O_4_ and evaluate its anti-cancer effects for HCC *in vitro*. The AuNC@Fe_3_O_4_ showed the typical small size of about 55 nm. Additionally, it demonstrated a high photothermal conversion efficiency and POD-like activity. The CCK-8 results demonstrated that AuNC@Fe_3_O_4_ had good biocompatibility and HCC cell-killing ability. Moreover, AuNC@Fe_3_O_4_ could synergistically stimulate cell death or apoptosis. Finally, it was observed that magneto-gold nanocomposites could facilitate 808 nm laser irradiation to increase their catalytic ability to produce ROS. This might promote lysosomal impairment, causing cell death or apoptosis. These results suggested that the AuNC@Fe_3_O_4_ may offer a promising anti-cancer strategy for HCC *via* the synergistic effect of PTT and nano-catalytic therapy. Further research is required to investigate the therapeutic efficacy of AuNC@Fe_3_O_4_ for HCC *in vivo*.

## Data Availability

The original contributions presented in the study are included in the article/Supplementary Material, further inquiries can be directed to the corresponding author.
