# Supplementary material for: A peroxidase-like magneto-gold nanozyme AuNC@Fe3O4 with photothermal effect for induced cell apoptosis of hepatocellular carcinoma cells in vitro
Source: Front Bioeng Biotechnol. 2023 Mar 23;11:1168750. doi: 10.3389/fbioe.2023.1168750 (PMC10076705; doi:10.3389/fbioe.2023.1168750)
Supplement: Supplementary file 1 [file Table1.pdf]

Table S1. The statistics of HepG2 cells with different treatments by flow cytometry.

Gate / Treatment	Q1 (%)	Q2 (%)	Q3 (%)	Q4 (%)
PBS-1	1.4	3.63	10.7	84.3
PBS-2	1.92	3.11	10.2	84.7
PBS-3	2.09	2.88	10.1	84.9
PBS+NIR-1	3.32	4.21	11	81.5
PBS+NIR-2	3.29	3.1	9.07	84.5
PBS+NIR-3	2.94	4.47	11.6	81
NC-1	6.26	12.4	16.7	64.7
NC-2	6.73	15.1	18.1	60.1
NC-3	7.36	14.4	17.7	60.6
NC+NIR-1	6.91	29.9	21.3	41.8
NC+NIR-2	8.17	29.6	21.2	41
NC+NIR-3	8.85	30.1	21.4	39.6
