# Supplementary material for: A peroxidase-like magneto-gold nanozyme AuNC@Fe3O4 with photothermal effect for induced cell apoptosis of hepatocellular carcinoma cells in vitro
Source: Front Bioeng Biotechnol. 2023 Mar 23;11:1168750. doi: 10.3389/fbioe.2023.1168750 (PMC10076705; doi:10.3389/fbioe.2023.1168750)
Supplement: Supplementary file 2 [file Image4.pdf]

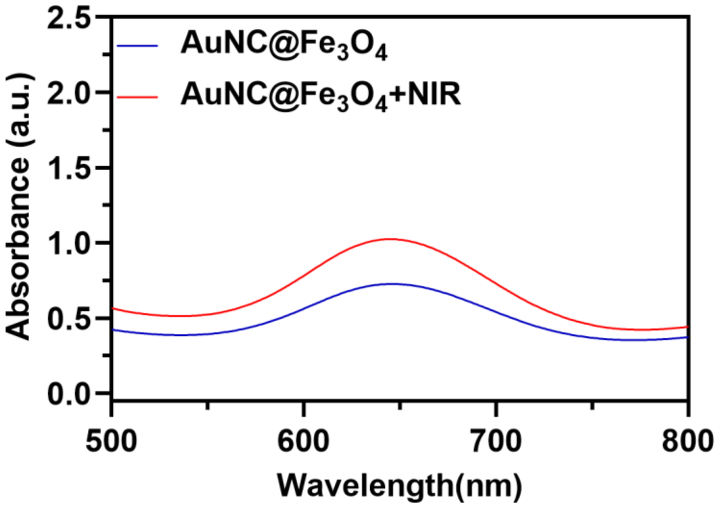

Supplementary Figure S4. The absorbance of solution with or without NIR irradiation was detected at 652 nm.
