# Supplementary material for: A peroxidase-like magneto-gold nanozyme AuNC@Fe3O4 with photothermal effect for induced cell apoptosis of hepatocellular carcinoma cells in vitro
Source: Front Bioeng Biotechnol. 2023 Mar 23;11:1168750. doi: 10.3389/fbioe.2023.1168750 (PMC10076705; doi:10.3389/fbioe.2023.1168750)
Supplement: Supplementary file 4 [file Image3.pdf]

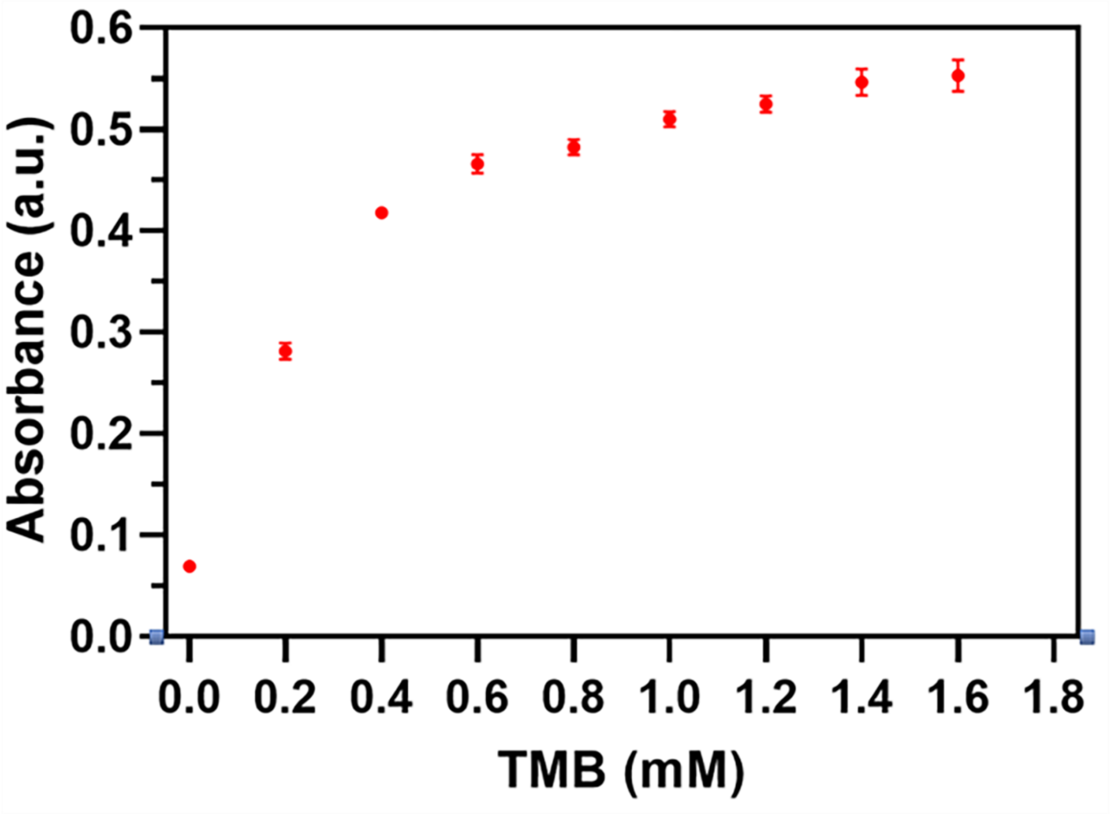

Supplementary Figure S3. The absorbance of solution was detected at 652 nm with varying TMB concentrations.
